# Adaptation of *Ustilago maydis* to phenolic and alkaloid responsive metabolites in maize B73

**DOI:** 10.3389/fpls.2024.1369074

**Published:** 2024-07-19

**Authors:** Xuanyue Guo, Zhen Yang, Jinjin Zhang, Juan Hua, Shihong Luo

**Affiliations:** Engineering Research Center of Protection and Utilization of Plant Resources, College of Bioscience and Biotechnology, Shenyang Agricultural University, Shenyang, China

**Keywords:** *Ustilago maydis*, maize B73, specialized metabolites, phytohormones, phenolic acids, alkaloids

## Abstract

The adaptation of pathogenic fungi to plant-specialized metabolites is necessary for their survival and reproduction. The biotrophic fungus *Ustilago maydis* can cause maize smut and produce tumors in maize (*Zea mays*), resulting in reduced maize yield and significant economic losses. Qualitative analysis using UPLC-MS/MS revealed that the infection of maize variety B73 with *U. maydis* resulted in increased levels of phytohormones, phenolics, and alkaloids in maize seedling tissues. However, correlation analysis showed that nearly all compounds in the mechanical damage group were significantly negatively correlated with the shoot growth indexes of maize B73. The correlation coefficients of 2-hydroxy-7-methoxy-1,4-benzoxazin-3-one (HMBOA) and maize B73 shoot length and shoot weight were *r* = -0.56 (*p* < 0.01) and *r* = -0.75 (*p* < 0.001), respectively. In the inoculation group, these correlations weakened, with the correlation coefficients between HMBOA and maize B73 shoot length and shoot weight being *r* = 0.02 and *r* = -0.1, respectively. The correlation coefficients between 6-methoxy-2-benzoxazolinone (MBOA) and the shoot weight were *r* = -0.73 (*p* < 0.001) and *r* = -0.15 in the mechanical damage group and inoculation group, respectively. These findings suggest that increased concentrations of these compounds are more positively associated with mechanical damage than with *U. maydis* infection. At high concentrations, most of these compounds had an inhibitory effect on *U. maydis*. This study investigated the ability of *U. maydis* to regulate various compounds, including phytohormones, phenolic acids, and alkaloids in maize B73, providing evidence that *U. maydis* has adapted to the specialized metabolites produced by maize B73.

## 1 Introduction

Plants increase production of substances, such as specialized metabolites, that provide direct or indirect defense following infection with pathogenic fungi (Weng et al., 2021; Geisen et al., 2022; Zhou et al., 2022). For example, upon infection with *Alternaria alternata*, tobacco will synthesize coumarins or sesquiterpenoids to enhance its resistance (Duan et al., 2016), and members of the Poaceae can initiate an effective defense mechanism by synthesizing alkaloids following infection by *Epichloë* sp (Bastías et al., 2022). However, increased production of specialized metabolites does not necessarily mean that plants have successfully defended themselves against pathogenic fungi. Even though there are many specialized metabolites in plants to resist external stress, pathogenic fungi can still successfully infect plants and cause disease (Bindics et al., 2022). This demonstrates that some specialized metabolites are not effective in plant defenses against fungi. For example, it has been reported that 2,4-dihydroxy-7-methoxy-2H-1,4-benzoxazin-3-one (DIMBOA) levels were significantly higher in tumor tissues following a fungal infection than in uninfected tissues (Basse, 2005). DIMBOA, a hydroxamic acid, is known for its role in maize seedling defense against various fungal pathogens (Frey et al., 1997). However, interestingly, the successful infection of maize by this fungus suggests that the fungus may have evolved the ability to adapt to DIMBOA. Following infection with pathogenic fungi, most plants have difficulties synthesizing compounds that directly inhibit the fungi. When subjected to stress, maize seedlings allocate a large amount of energy to the activation of the defense system (Babikova et al., 2013). The levels of many alkaloids, including the benzoxazolone derivative MBOA, significantly increased in maize plants infected with the pathogenic fungus *Fusarium verticillioides* (Glenn et al., 2002), although the increase in the levels of these compounds did not prevent the infection of *F. verticillioides*. Thus, the ability of pathogenic fungi to adapt to or detoxify host defense substances is the key to the successful infection of their hosts (Spaepen et al., 2007; Djamei et al., 2011; Lanver et al., 2017). Indeed, fungi have evolved diverse adaptive mechanisms to survive and reproduce on plants, and some species can convert and metabolize specialized plant compounds and use them to promote their growth. Phloridin is one of the main metabolites of *Malus*. Our previous research found that *Valsa mali* var. *mali*, a fungus that infects branches of apple trees, can utilize phlorizin to produce a series of degradation products, promoting its germination and allowing the fungus to successfully infect its host species (Yang et al., 2022). Investigation of the mechanisms by which fungi can adapt to or degrade plant secondary metabolites remains a hot topic worthy of further study.

The biotrophic fungus *Ustilago maydis* can cause smut disease in all organs of its host plant, maize, and is primarily known to cause tumors (Kahmann and Kämper, 2004; Wu et al., 2023). Its initial infection usually occurs in plant seedlings (Redkar et al., 2015), and infected leaf surfaces develop invagination. The epidermal and subepidermal cells of the leaves are then penetrated by the fungus and form an interaction zone where the mycelium is encapsulated in the host plasma membrane (Okmen and Doehlemann, 2014; Matei et al., 2018). The fungal mycelium then proliferates rapidly at the infected site, causing the infected maize plants to lose their green color (yellowing of tissues) (Basse, 2005) as the levels of chlorophyll gradually decrease. This leads to the significant accumulation of anthocyanins in the infected area and the promotion of the progression of infection if the plants are exposed to light (Tanaka et al., 2014). Tumors typically appear between 5 days to 7 days post-infection due to the proliferation of both host and fungal cells (Okmen and Doehlemann, 2014; Matei and Doehlemann, 2016). These manifestations on maize plants become rapidly obvious after infection, and the infection of maize by *U. maydis* can be described as “fast, accurate, and ruthless.” The time between the initial infection of maize by *U. maydis* and the appearance of tumors is usually only 1 week. Infestation of the cob by *U. maydis* can lead to a reduction in yield in maize and cause significant economic losses (Brefort et al., 2009). Fungal infection causing plant disease in crop plants is widespread and seriously endangers food safety (Fisher et al., 2012), and the mechanisms by which it can infect its host plants have become a hot topic in the study of plant-fungal interactions.

In this study, we performed qualitative and quantitative analyses of the phytohormones, phenolics, and alkaloids in *U. maydis*-infected maize to identify the chemicals involved in the response of maize seedlings to *U. maydis* infection. Further correlation analysis combined with activity screening revealed how *U. maydis* can “smartly” adapt to the defense system of the host and eventually succeed in parasitizing the host.

## 2 Materials and methods

### 2.1 The fungus *U. maydis* and plant materials

The biotrophic fungus (*Ustilago maydis*, SG200) was provided by the College of Agriculture, Shenyang Agricultural University. The SG200 strain was cultured in potato dextrose agar (PDA) medium (1 L of deionized water containing 20 g of glucose and 15 g of agar) at 28°C for 2 days. Maize B73 seeds were obtained through the self-pollination of maize plants in the experimental field at Shenyang Agricultural University (123°57′ E, 41°83′ N).

### 2.2 Inoculation of maize B73 with SG200 and monitoring of its growth and development

**Figure 1:**
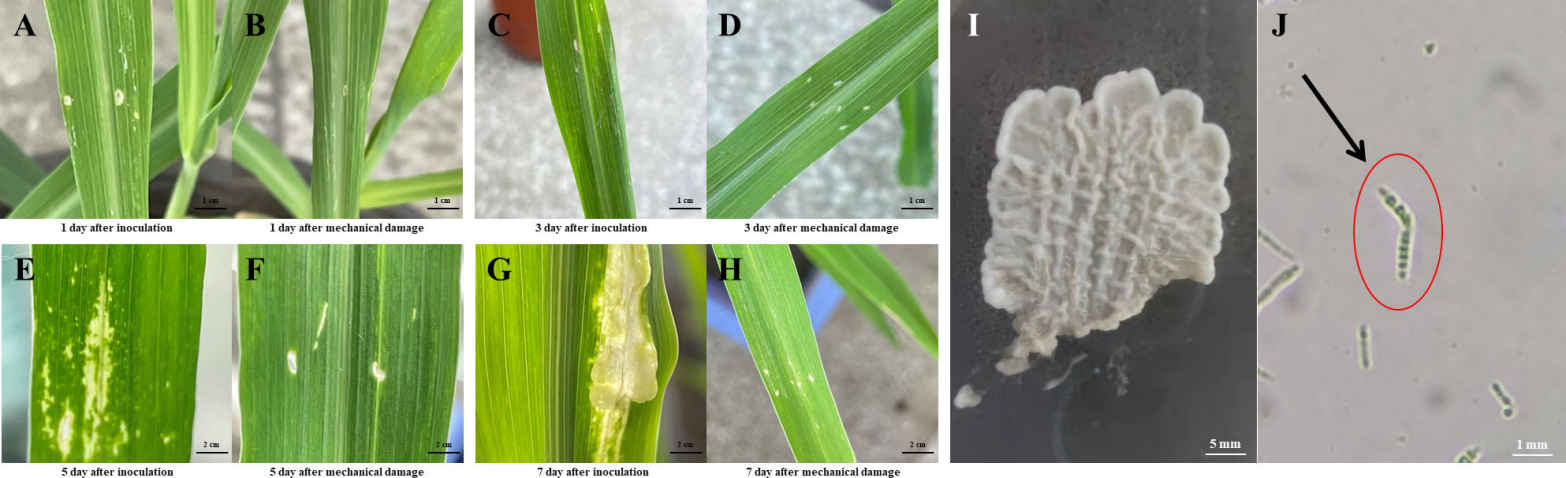
Biological morphology of *U. maydis* and tumor formation in maize following infection. **(A-H)** Growth status of the inoculation and mechanical damage groups following inoculation with *U. maydis*. **(I)** Morphology of *U. maydis* in PDA solid medium. **(J)** *U. maydis* spore observed under a stereo microscope. Scale bar: **(A-D)**, 1 cm; **(E-H)**, 2 cm; **(I)**, 5 mm; **(J)**, 1 mm.

SG200 was cultured for no longer than 1 week in a PDA medium to activate it (**Figure 1I**). Then, periphery colonies of SG200 were selected from the PDA medium, inoculated into 3 mL of yeast extract peptone and sucrose L (YEPSL) medium, and incubated at 28°C at 200 rpm overnight (Zuo et al., 2023). Multiple repeats were performed to ensure the purity of the strains and the success rate of activation. After the medium became turbid, 40 μL was added to 50 mL of YEPSL medium in a 250 mL Erlenmeyer flask and shaken at 200 rpm at 28°C for 12 h. The culture was collected when the optical density reached 0.6–0.8 at 600 nm (OD_600_), and this OD range ensures the growth phase of the *U. maydis* during the active dividing phase. The collected culture was centrifuged (4528 g/5 min) to remove cells from the medium, and the pellet was washed three times with sterile water. SG200 was then resuspended in sterile water until the OD_600_ = 1.0, at which point the spore suspension was used to infect maize plants. For plant inoculations, 300 μL of SG200 spore suspension was inoculated onto the meristematic zone of V3 (third leaf) stage maize using a 1 mL sterile syringe (**Supplementary Figure S1**). At this time, the syringe should point to the center of the leaf whirl, and the injection site should be selected at the young stem site, about 1 cm from the soil. Successfully infected maize plants will develop tumors after 4–6 dpi (**Figure 1G**) (Redkar and Doehlemann, 2016). The mechanical damage group was injected with the same volume of sterile water as the control group to investigate the effect of mechanical damage on maize B73. The growing maize seedlings were then cultivated at a constant temperature of 28°C. Four growth indexes (root and shoot length and weight) were measured in maize seedlings inoculated with SG200 or sterile water as a control. At 1 day, 3 days, 5 days, 7 days, 9 days, 11 days, 13 days, and 15 days after inoculation, the maize B73 seedlings were taken and divided into shoot and root parts. The lengths and fresh weights of these parts were measured, with three replicates in each group.

### 2.3 Establishment of the calibration curve

The standard compounds IAA, 6BA, ABA, SA, HBA, MeP, VAE, *p*HB, MHPP, MBOA, HMBOA, DMBOA, NMC, DIBOA, and BOA were dissolved separately in chromatographic methanol, each with a final solution concentration of 5 μg/mL. All standard substances were purchased from professional merchandise suppliers. UPLC-MS/MS (LCMS-8050, Shimadzu Scientific Instruments, Inc., Tokyo, Japan) was used to find the precursor and product ions for each standard sample, as well as the conditions necessary for the multi-reaction monitoring mode (MRM) analysis (see **Supplementary Material** for the ion pairs of the compounds, **Supplementary Table S1**). The UPLC-MS/MS was used with the following parameters: heating gas flow, 5 L/min; interface temperature, 300°C; DL temperature, 250°C; desolvation line temperature, 250°C; heat block temperature, 450°C; and drying gas flow, 15 L/min. A total of 1 μL sample was injected into a Shim-pack GIST C_18_ column (2 μm, 100 × 2.1 mm) with a flow rate of 0.2 L/min, and the column temperature was maintained at 40°C. Compounds A (0.1% acid water) and B (acetonitrile) were used as the mobile phases. See **Supplementary Material** for the elution procedure (**Supplementary Table S2**).

Notably, 1 mg/mL solutions of the standard samples were prepared in chromatographic-grade methanol. The samples were then serially diluted to 5 μg/mL, 2 μg/mL, 1 μg/mL, 0.5 μg/mL, 0.2 μg/mL, 0.1 μg/mL, 0.05 μg/mL, 0.02 μg/mL, and 0.01 μg/mL. UPLC-MS/MS was used to calculate the peak response and to obtain the calibration curve, which can be used in quantitative calculations, for each sample. See the **Supplementary Material** for calibration curve equations (**Supplementary Table S3**).

### 2.4 Quantification of compounds in maize B73 after infection with *U. maydis*

Maize B73 seedlings were planted in a growth chamber in brunisolic soil previously sterilized by heating to 120°C for 30 min three times. All maize seedlings were grown in disposable seedling pots. Before sowing, 3/5 of the brunisolic soil was added to the pot. Three seeds were placed evenly on the surface, and then the pot was filled with brunisolic soil to 4/5 full. The brunisolic soil was originally collected from maize fields in the experimental field of Shenyang Agricultural University. Maize seeds were directly planted in pots as described above, then incubated in a growth chamber with a photoperiod of 16 h light/8 h dark at 24°C. When the maize seedlings had grown to the V3 (third leaf) stage, the spore suspension prepared above was used to infect the seedlings. Maize inoculated with an equivalent amount of water was used as the control group. Shoot and root samples were collected from the maize seedlings on days 1, 3, 5, 7, 9, 11, 13, and 15 following inoculations, with three replicates. During sampling, the aboveground parts (shoots) and the underground parts (roots) were distinguished. 0.5 g of plant materials were taken from each part to grind, then transferred into 10 mL capped centrifuge tubes. 4 mL of extraction buffer (methanol, water, and formic acid = 15: 4: 1) was added to each sample. The samples were incubated in an ultrasonic bath for 45 min, after which a further 4 mL of extraction buffer was added. After sonicating for 45 min, the samples were centrifuged (3578 g/10 min), and the supernatant was then carefully aspirated and purified on an HLB column to obtain the eluates. The eluates were collected and evaporated to complete dryness using rotary evaporation at 40°C. The final volume was made up to 1 mL with chromatographic -grade methanol, and the samples were then passed through 0.22 μm filters. This part of the sample was used for the analysis of alkaloids. For the analysis of phytohormones and phenolic acids, a 0.5 -g shoot or root sample from each group was weighed. The extraction was performed as above. After the liquids were purified on an HLB column, the remaining solution was passed through the MCX column, and the waste liquid was discarded (Jia et al., 2020). Subsequently, the column was eluted with methanol, and the eluate was collected and heated to 40°C until it was almost dry. The final volume was then prepared with chromatography-grade methanol to 1 mL through a 0.22 μm filter. Metabolites in the samples were analyzed using a UPLC-MS/MS on a Shim-pack GIST C_18_ column (2 μm, 100 × 2.1 mm) following previously described methods.

### 2.5 Assessment of germination of SG200 spores following treatment with specialized metabolites

A suspension of SG200 spores was prepared according to the method described above. Each specialized metabolite was weighed and dissolved in methanol, so the final concentration was 1024 μg/mL, after which 1 μL of the dissolved compound was added to 199 μL of PDB medium (0.5% of the total volume). The metabolites were then diluted to a range of values between 16 μg/mL and 512 μg/mL using a double dilution method. Subsequently, 100 μL of spore suspension and 100 μL of the PDB solution mixture containing different concentrations of metabolites were mixed in a 96-well plate. The negative control contained an equal volume of methanol without the specialized metabolite, while the positive control contained nystatin, and each treatment was repeated five times. The plates were sealed with parafilm and cultured at 28°C. The absorbance of all cultures was measured at 600 nm (OD_600_) every 12 h using a 96-well plate reader. Simultaneously, the germination of SG200 spores treated with these compounds was visually measured under a light microscope. When the length of the spore germ tube was greater than the radius of the short spore, we thought the spore germination was successful (Yang et al., 2022).

### 2.6 Statistical analyses

The data shown are the means ± SD across all biological replicates. The statistical analysis was conducted in the SPSS 12.0 statistical software package. Significant differences between the two groups of data were assessed using Student *t-*tests (*p value *t*-test < 0.05, **p value *t*-test < 0.01, ***p value *t*-test < 0.001). ANOVA and *post-hoc* Tukey tests comparing mean differences were used to analyze data from more than two groups (*p* < 0.05). The R program (www.r-project.org) and R Studio (ggplot2) were used for statistical analysis and calculations.

## 3 Results

### 3.1 The formation of tumors induced by *U. maydis* fungi

Following cultivation in a PDA medium, *U. maydis* strain SG200 formed pale yellow colonies on the PDA plate, with neat colony edges and several protrusion lines inside the colony (**Figure 1I**). After a week of growth, a little of the mycelium at the edge was scraped away and diluted with sterile water on a slide. The spore morphology was observed under a stereo microscope, and it was found that the spores of *U. maydis* showed an elongated shape (**Figure 1J**).

*U. maydis* was inoculated onto the leaves of B73 maize seedlings to observe the formation of tumors on the maize leaves (**Figures 1A–H**). Approximately a week following inoculation, the maize seedlings in the inoculation group showed significant tumor formation, while the seedlings in the mechanical damage group showed no significant changes. On the first day (**Figures 1A, B**) and the third day (**Figures 1C, D**) following maize infection with *U. maydis*, there were no visual differences between the inoculation and the mechanical damage groups. However, on the fifth day after inoculation, the infected plants (**Figure 1E**) had lost their green color and were turning yellow. By day 7, tumor formation had been observed in the inoculation group of maize seedlings (**Figure 1G**). The green area surrounding the tumor tissues decreased, and more tissue chlorosis occurred as the thickness of the tumor tissues increased. A small area of protrusions also appeared at this time on the maize seedlings, indicating the successful infection of *U. maydis*. No changes in appearance were observed in the mechanical damage group (**Figure 1H**).

### 3.2 *U. maydis* affected the growth phenotype of seedling maize

**Figure 2:**
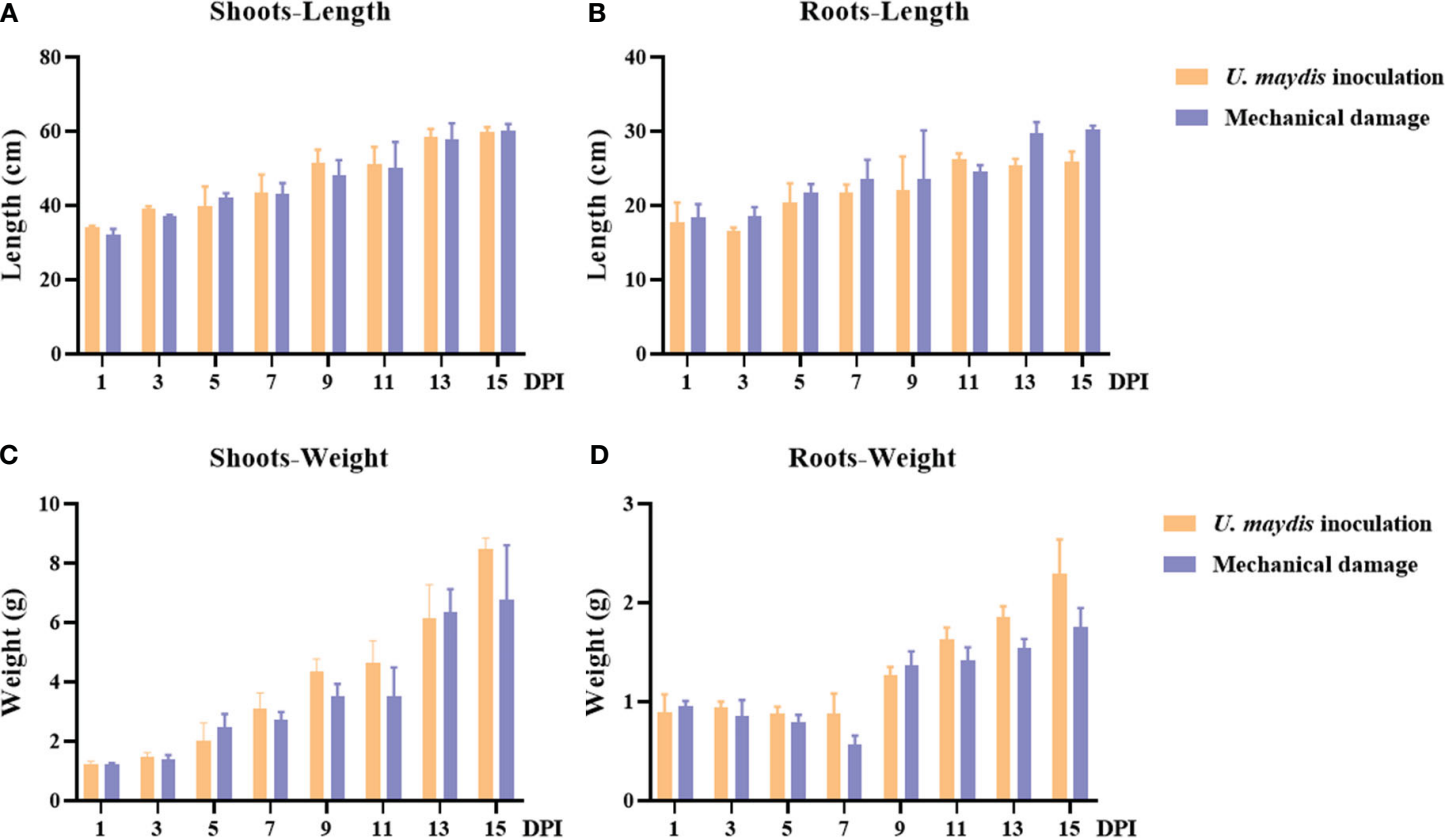
Phenotype determination of seedling maize following inoculation with *U. maydis* SG200. **(A, B)** Maize shoot and root length during the 15 days following inoculation with *U. maydis*. **(C, D)** Maize shoot and root weight during the 15 days following inoculation with *U. maydis*. The data shown are means ± SD, and the error bars in all graphs indicate the standard deviation across all biological replicates.

The length and weight of maize B73 shoots increased with time, but there was no significant difference observed between the inoculation and the mechanical damage groups (**Figures 2A–D**). The shoot weight ranges of these two groups were 1.13 g–8.74 g and 1.20 g–8.89 g, respectively (**Figure 2C**). The weights of the shoots reached their highest values on day 15 after inoculation, with no significant difference in mean weight between the two groups (inoculation group, 8.48 g; mechanical damage group, 6.75 g).

The length and weight of maize B73 roots were also found to increase over time (**Figures 2B, D**). However, by the 15^th^ day, the root weight of the inoculation group (2.30 ± 0.33 g) was different from that of the mechanical damage group (1.75 ± 0.19 g). At this time, the mean length of the roots in the inoculation group was 25.97 ± 1.28 cm, while the length of the roots in the mechanical damage group was 30.27 ± 0.48 cm. The observed differences between the inoculation and mechanical damage groups at specific times demonstrate that *U. maydis* infection affected the growth indexes of maize B73 seedlings.

### 3.3 The inoculation with *U. maydis* affects levels of phytohormones in maize seedlings

**Figure 3:**
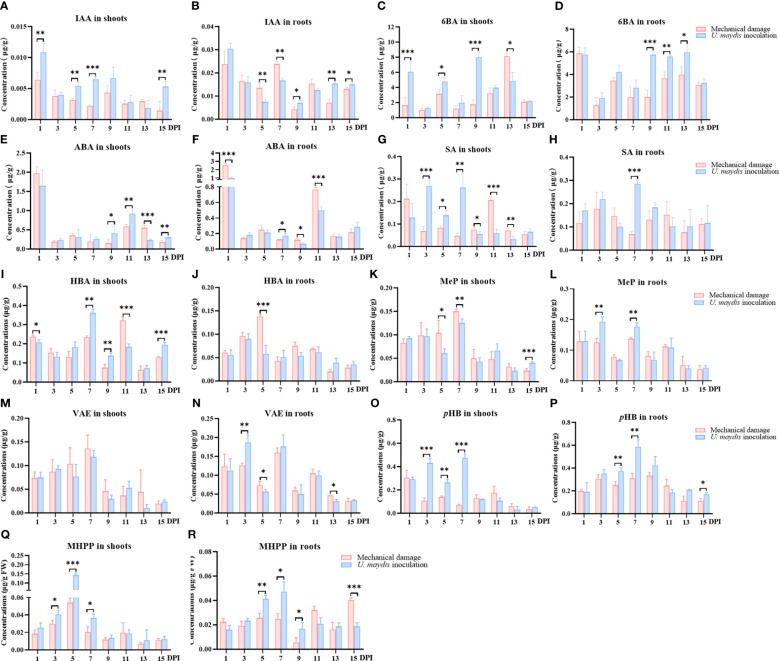
Quantitative analyses of phytohormones and phenolic acids in maize seedlings infected with *U. maydis*. Concentrations of IAA **(A, B)**, 6BA **(C, D)**, ABA **(E, F)**, SA **(G, H)**, HBA **(I, J)**, MeP **(K, L)**, VAE **(M, N)**, *p*HB **(O, P)**, and MHPP **(Q, R)** in maize shoots and roots during the 15 days following inoculation. IAA, indole-3-acetic acid; 6BA, 6-benzylaminopurine; ABA, abscisic acid; SA, salicylic acid; HBA, 4-OH-benzaldehyde; MeP, methyl 4-OH-benzoate; VAE, vanillin; *p*HB, *p*-hydroxybenzoic acid; MHPP, methyl 4-hydroxycinnamate. The data shown are means ± SD, and the error bars in all graphs indicate the standard deviation across all biological replicates. One, two, or three asterisks (*, **, or ***) indicate significant differences (at *p* < 0.05, *p* < 0.01, or *p* < 0.001, respectively) between the controls and the other treatments at the same time, as determined using Student *t*-tests.

To determine the effect of *U. maydis* on the levels of specialized metabolites in maize, UPLC-MS/MS was used to quantitatively analyze the levels of phytohormones in maize B73 seedlings over the 15 days following inoculation with *U. maydis* (**Figures 3A–H**). In total, 15 phytohormones in maize B73 were quantitatively analyzed. The levels of four phytohormones (indole-3-acetic acid (IAA), 6-benzylaminopurine (6BA), abscisic acid (ABA), and salicylic acid (SA)) were significantly different in the maize seedlings over the 15 days following treatment. The other tested phytohormones showed no significant differences between test groups over the time investigated. Concentrations of IAA, 6BA, and SA in the shoots of the inoculation group were higher than those in the mechanical damage group within the first 7 days following infection in shoots (**Figures 3A, C, G**) . On day 5, the concentrations of these three compounds in the inoculation group were significantly higher than those in the mechanical damage group, with concentrations of IAA in the inoculation and mechanical damage groups being 0.005 ± 0.001 μg/g FW and 0.03 ± 0.01 μg/g FW (*p* < 0.01), respectively. The concentrations of 6BA were 4.79 ± 0.55 μg/g FW and 3.17 ± 0.55 μg/g FW (*p* < 0.05). The concentrations of SA in the inoculation and mechanical damage groups were 0.14 ± 0.02 μg/g FW and 0.08 ± 0.01 μg/g FW (*p* < 0.05), respectively. On days 9 and 11, following inoculation, the concentration of ABA was also higher in the shoots of the inoculation group than those of the mechanical damage group.

The responses of some maize phytohormones in response to *U. maydis* infection were slightly later in roots than those in shoots and did not become significant until the seventh or ninth day. SA levels in the inoculation group were significantly higher than in the mechanical damage group 7 days following treatment (*p* < 0.001) (**Figure 3H**). From the 9th day to the 15th day, IAA and 6BA in maize roots also responded positively to the infection of *U. maydis*. The concentration of 6BA in the roots of the inoculation group was 5.74 ± 0.28 μg/g FW on the ninth day following infection, while that in the roots of the mechanical damage group was 2.00 ± 0.61 μg/g FW (*p* < 0.001) (**Figure 3D**). These results confirmed that the concentrations of different phytohormones in maize B73 increased following infection with *U. maydis* SG200. It also may explain the observed root and shoot length and weight increases in the maize seedlings following infection with *U. maydis*.

### 3.4 Inoculation with *U. maydis* affects levels of phenolic acids in maize seedlings

UPLC-MS/MS was used to quantitatively analyze the levels of phenolic acids in maize B73 seedlings following inoculation with *U. maydis* (**Figures 3I–R**). In total, 11 phenolic acids were quantitatively analyzed. The levels of 4-OH-benzaldehyde (HBA), methyl 4-OH-benzoate (MeP), vanillin (VAE), *p*HB, and methyl 4-hydroxycinnamate (MHPP) varied irregularly over the 15 days following inoculation in the treatment group, although all reached their maximum levels on the fifth or seventh day after inoculation. The levels of other tested phenolic acids were not significantly different between the inoculation and mechanical damage groups. In the shoots, the levels of VAE, MHPP, and MeP showed an approximately parabolic trend with increasing time following inoculation. The concentrations of MHPP in the inoculation and mechanical damage groups 5 days following infection were 0.14 ± 0.05 μg/g FW and 0.05 ± 0.01 μg/g FW, respectively, with levels in the inoculation group being significantly higher than those in the mechanical damage group (*p* < 0.001) (**Figure 3Q**). Interestingly, *p*HB showed significantly higher concentrations in maize shoots in the inoculation group than in the mechanical damage group on the third, fifth, and seventh days after infection (**Figure 3O**).

In the roots, the concentration of *p*HB in the inoculation group showed an obvious trend, increasing first and then decreasing (**Figure 3P**). Concentrations ranged from 0.16 μg/g FW to 0.65 μg/g FW in the inoculation group and only 0.10 μg/g FW to 0.36 μg/g FW in the mechanical damage group. On the seventh day after inoculation, the highest concentration was 0.59 ± 0.06 μg/g FW in the inoculation group and only 0.31 ± 0.04 μg/g FW in the mechanical damage group (*p* < 0.01).

### 3.5 Inoculation with *U. maydis* affects levels of alkaloid -specialized metabolites in maize seedlings

**Figure 4:**
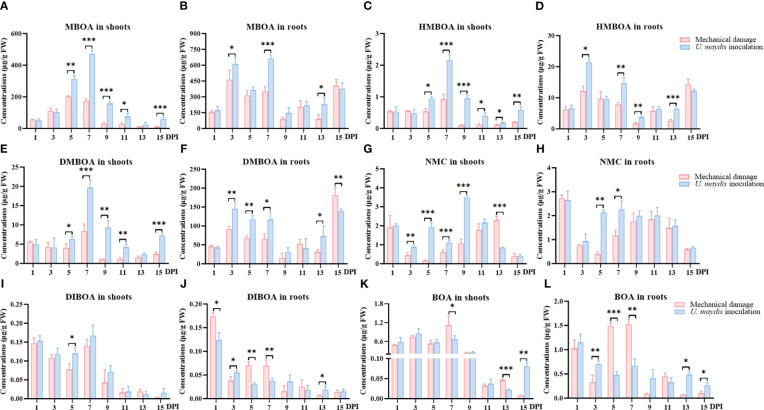
Quantitative analysis of specialized metabolites in maize seedlings infected with *U. maydis*. Concentrations of MBOA **(A, B)**, HMBOA **(C, D)**, DMBOA **(E, F)**, NMC **(G, H)**, DIBOA **(I, J)**, and BOA **(K, L)** in shoots and roots during the 15 days following inoculation. MBOA, 6-methoxy-benzoxazolin-2-one; HMBOA, 2-hydroxy-7-methoxy-1,4-benzoxazin-3-one; DMBOA, 6,7-dimethoxy-2-benzoxazolimone; NMC, N-methyl-6-methoxybenzoxazolinone; DIBOA, 2,4-dihydroxy-1,4-benzoxazin-3-one; BOA, 2-benzoxazolin-2 (3H)-one. The data shown are mean ± SD, and the error bars in all graphs indicate the standard deviation across all biological replicates. One, two, or three asterisks (*, **, or ***) indicate significant differences (at *p* < 0.05, *p* < 0.01, or *p* < 0.001, respectively) between the controls and the other treatments at the same time, as determined using Student *t*-tests.

The levels of six alkaloid metabolites MBOA, HMBOA, 6,7-dimethoxy-2-benzoxazolimone (DMBOA), *N*-methyl-6-methoxybenzoxazolinone (NMC), 2,4-dihydroxy-1,4-benzoxazin-3-one (DIBOA), and 2-benzoxazolin-2 (3*H*)-one (BOA) were quantitatively analyzed in maize seedlings using UPLC-MS/MS (**Figures 4A–L**) over the 15 days following infection with *U. maydis*.

The responses of these specialized metabolites to the inoculation were varied. In the maize shoots, MBOA, HMBOA, and DMBOA showed a relatively uniform trend, with their concentrations gradually increasing after inoculation until the 7th day, and then gradually decreasing to the 13th day. In addition, between the 5th and the 15th day following inoculation, levels of these chemicals in the treatment group were also significantly higher than in the mechanical damage group. The responses of NMC, DIBOA, and BOA to the inoculation were not strongly positive, and the concentrations even decreased in the inoculation group in a few periods.

The concentrations of MBOA, HMBOA, and NMC in maize roots in the inoculation group were significantly higher than those in the mechanical damage group during much of the assessment period. Seven days following inoculation, the concentration of MBOA in the inoculation group was 663.17 ± 37.18 μg/g FW, while the concentration in the mechanical damage group was 352.41 ± 43.08 μg/g FW (*p* < 0.001) (**Figure 4B**). However, the concentration of DIBOA in the maize roots was significantly lower in the inoculation group than in the mechanical damage group (**Figure 4J**).

### 3.6 Correlation analyses between maize seedling growth indexes and the levels of phenolic acids or alkaloids

**Figure 5:**
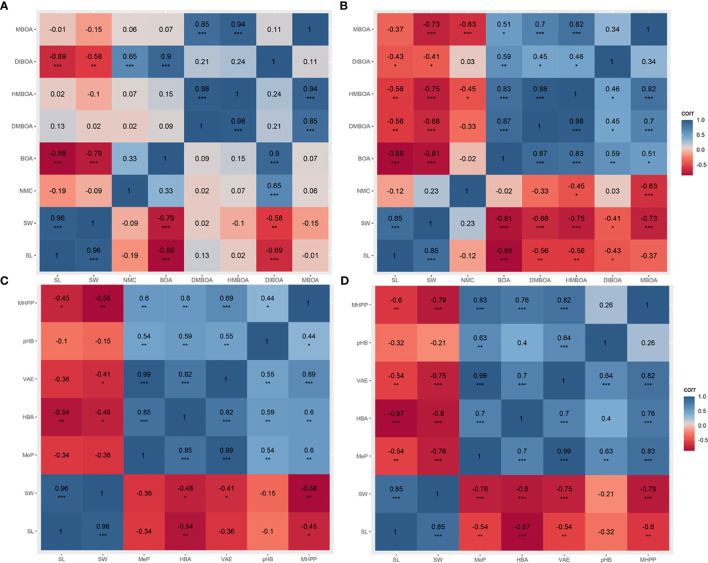
Correlation analysis between maize shoot growth indexes and specialized metabolites. Correlation analysis between length, weight, and alkaloids of the inoculation group **(A)** and the mechanical damage group **(B)**. Correlation analysis between length, weight, and phenolic acids of the inoculation group **(C)** and the mechanical damage group **(D)**. SL, shoot length; SW, shoot weight. The blue and red squares represent positive and negative correlations, respectively. One, two, or three asterisks (*, **, or ***) indicate significant differences (at *p* < 0.05, *p* < 0.01, or *p* < 0.001, respectively) between the controls and the other treatments at the same time, as determined using Student *t*-tests.

Correlation analyses were run between the indicators representing the growth of maize B73 seedlings (length and weight) and either alkaloid metabolites in the seedlings (**Figures 5A, B**) or phenolic acid metabolites (**Figures 5C, D**). In maize B73 seedling shoots, a significant negative correlation was observed between almost all compounds and maize growth indices in the mechanical damage group, whereas these correlations were attenuated in the inoculation group (**Figures 5A, B**). DMBOA, HMBOA, and MBOA showed consistent performance in the inoculation group; no significant correlations between the three compounds and maize growth were observed. However, these three compounds showed a stronger and more significant negative correlation with the growth length and weight of maize in the mechanical damage group. The correlation coefficients between these three compounds and shoot weight in the inoculation group were *r* = 0.02, *r* = -0.10, and *r* = -0.15, respectively (**Figure 5A**). However, the correlation coefficients between these three compounds and shoot weight in the mechanical damage group were *r* = 0.-68, *r* = -0.75, and *r* = -0.73, respectively, with *p* < 0.001 (**Figure 5B**). NMC was not correlated with maize growth indexes in either the inoculation or the mechanical damage group. However, BOA and DIBOA had the same correlation with the maize shoot length following either *U. maydis* infection or mechanical damage. In maize B73 seedling roots, there were no obvious correlations between any of the tested compounds and the growth indexes (**Supplementary Figures S2A, B**).

Correlation analyses were also conducted between specialized phenolic metabolites and the weight of maize B73 seedling growth indexes (**Figures 5C, D**). In maize shoots, the correlations between the tested phenolics and the growth indexes in the inoculation group were weaker than those in the mechanical damage group (**Figures 5C, D**). Specifically, in the inoculation group, the correlation coefficient between MeP and shoot length was *r* = -0.34, and that between MeP and shoot weight was *r* = -0.36. In the mechanical damage group, the correlation coefficient between MeP and shoot length was *r* = -0.54 (*p* < 0.01), and that between MeP and shoot weight was *r* = -0.76 (*p* < 0.001). Moreover, the three compounds HBA, VAE, and MHPP showed a higher correlation with the maize growth indexes in the mechanical damage group than in the inoculation group. However, *p*HB was correlated with neither shoot length nor weight in either group. The correlation coefficients between *p*HB and shoot length and weight in the inoculation group were *r* = -0.1 and *r* = -0.15, respectively (**Figure 5C**), and those between *p*HB and shoot length and weight in the mechanical damage group were *r* = -0.32 and *r* = -0.21, respectively (**Figure 5D**). In the roots, the correlations between the phenolic compound concentrations and maize growth indexes were more complex (**Supplementary Figures S1C, D**).

### 3.7 Effects of maize -specialized metabolites on the germination of *U. maydis* spores

**Figure 6:**
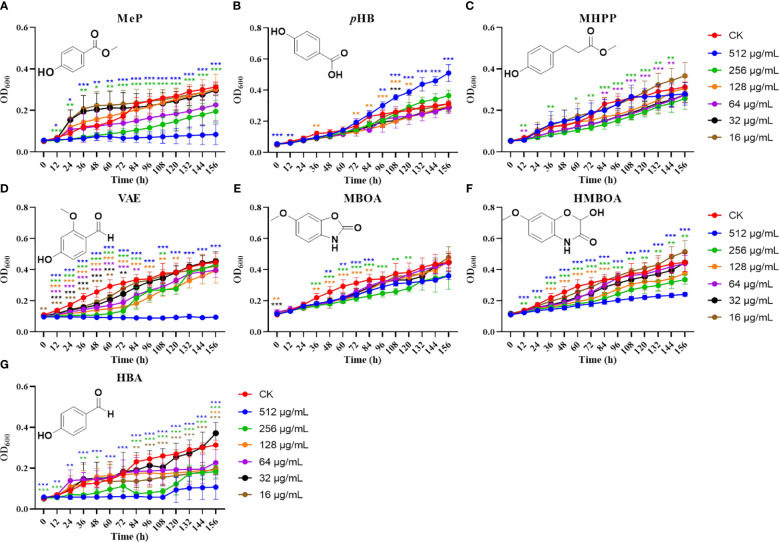
Effects of different metabolites on *U. maydis* spore germination. Effects of different concentrations of MeP **(A)**, *p*HB **(B)**, MHPP **(C)**, VAE **(D)**, MBOA **(E)**, HMBOA **(F)**, and HBA **(G)** on *U. maydis* spore germination. The data shown are means ± SD, and the error bars in all graphs indicate the standard deviation across all biological replicates. One, two, or three asterisks (*, **, or ***) indicate significant differences (at *p* < 0.05, *p* < 0.01, or *p* < 0.001, respectively) between the controls and the other treatments at the same time, as determined using Student *t*-tests.

To investigate the effects of increasing concentrations of specialized metabolites from maize on the germination of *U. maydis* spores, MBOA, HMBOA, and the phenolic acids listed above were added to *U. maydis* spore cultures with 1/2 PDB for 156 h (**Figures 6A–G**).

In most cases, the OD_600_ value gradually increased with time. However, the effects of different compounds on the germination rate of *U. maydis* spores were different. At high concentrations, most of the tested compounds inhibited *U. maydis* spore germination. After 156 h of co-culture, OD_600_ values of 512 μg/mL HMBOA ranged from 0.11 ± 0.004 to 0.24 ± 0.012, while the OD_600_ values of the control group ranged from 0.11 ± 0.005 to 0.45 ± 0.060 (**Figure 6F**). From 12 h to 156 h, the treatment group of 512 μg/mL HMBOA was significantly higher than the control group (*p* < 0.001). Furthermore, at concentrations of 128 μg/mL and 256 μg/mL, HMBOA also inhibited spore germination to different degrees. The inhibitory effect on spore germination at high concentrations is also seen in MeP, MHPP, VAE, MBOA, and HBA, and the higher the concentration of each of these compounds, the more inhibited the germination rate of *U. maydis* spores.

In contrast to the other tested compounds, high concentrations of *p*HB were able to promote the growth of *U. maydis* spores (**Figure 6B**). The OD_600_ values of 512 μg/mL *p*HB ranged from 0.06 ± 0.001 to 0.51 ± 0.054, which showed a significant difference from the control group starting at 96 h (*p* < 0.01). At 156 h, the OD_600_ value of 512 μg/mL *p*HB was 0.51 ± 0.054, while that of the control group was only 0.31 ± 0.023 (*p* < 0.001). These results indicate that a high concentration of *p*HB can promote the germination of *U. maydis* spores. Although there was no significant *p*HB response to *U. maydis* infection or mechanical damage in the correlation analysis, quantitative analysis following *U. maydis* infection found that concentrations of *p*HB in maize were significantly upregulated in response to *U. maydis*.

## 4 Discussion

Phytohormones are trace compounds produced by plants to regulate their physiological processes and are particularly important for plant growth (Chen et al., 2023). The link between tumor expansion caused by *U. maydis* infection and plant cell proliferation indicates the involvement of phytohormones (Doehlemann et al., 2008). It has been suggested that cytokinin (CTK) levels are altered in maize plants after infection by the *U. maydis* pathogen (Argueso et al., 2009). It has also been hypothesized that *U. maydis* can produce IAA to induce host tumor development, and indeed, IAA concentrations in tumor tissues were 20-fold and 5-fold higher than in uninfected maize stalk tissues before and during sporulation, respectively (Reineke et al., 2008). These results suggest that *U. maydis* may be able to change the levels of phytohormones in maize seedlings. IAA is a major growth factor in plants and has an important role in regulating plant growth and development, including cell division, differentiation, and elongation (Galli et al., 2015). On the fifth day after infection with *U. maydis*, maize B73 began to fade green and yellow, then formed tumors. These results demonstrate that the infection of seedling maize by *U. maydis* can significantly change the physiological characteristics and appearance of the maize plants. The lengths and weights of both shoots and roots of maize B73 increased during the early stages of infection, suggesting that *U. maydis* may promote the growth of maize B73 over a certain period. Therefore, phytohormones such as IAA may be involved in the short-term increased cell growth associated with tumor formation following *U. maydis* infection. Phenolics have been reported to promote plant defense by converting into active defense chemicals in damaged plant tissues (Navarro et al., 2008). The phenolic acid compounds were also affected by *U. maydis*. However, combining the results from the shoots and roots, *p*HB concentration increased significantly after maize B73 was infected by *U. maydis*, indicating that it may be produced in response to *U. maydis* infection.

Many studies have shown that specialized metabolites have a defensive role in plants (Hunziker et al., 2021; Zeng et al., 2021; Ma et al., 2022). Plants can activate their defense systems to produce higher concentrations of specialized metabolites such as alkaloids in response to external stress, particularly following biological stress (Speed et al., 2015; Marone et al., 2022). Benzoxazines and terpenoids are important defense chemicals in plants and may have been isolated and characterized (Li et al., 2021). The concentrations of alkaloid -specialized metabolites in maize B73 seedlings increased after inoculation with *U. maydis*, indicating activation of the endogenous defense system of the plant in response to pathogenic invasion. However, not all compounds exhibited discernible effects. Of the tested compounds, the concentrations of DMBOA, NMC, DIBOA, and BOA in the inoculation group were occasionally significantly lower than those in the mechanical damage group in shoots or roots. However, MBOA and HMBOA both consistently showed significantly higher concentrations in the inoculation group than in the mechanical damage group, indicating that these two compounds continued to respond to *U. maydis* infection during this period. These results indicate that maize increases the tissue concentrations of MBOA and HMBOA over a certain period in response to infection with *U. maydis*. Subsequent correlation analysis showed that the specialized metabolites in the mechanical damage group were significantly negatively correlated with the maize growth indexes in the shoots, indicating that these compounds were produced in response to the stress of mechanical damage. Nevertheless, the weakened correlation of the inoculation groups in the correlation analysis indicated that inoculated *U. maydis* did not have a strong activation effect on these compounds. Most of the compounds tested here had inhibitory effects on the germination of *U. maydis* spores, which means they could have a role in defense in maize B73. Nonetheless, the results of the correlation analysis indicate that the concentrations of these compounds are more strongly correlated with mechanical damage than with *U. maydis* infection. Indeed, the *U. maydis* infection weakens these correlations. Therefore, even though the quantitative results suggested that two compounds (MBOA and HMBOA) were produced strongly in response to the inoculation of *U. maydis*, these substances were not able to inhibit *U. maydis* infection.

We know that *U. maydis* is a worldwide maize disease (Yu et al., 2023). Studies on Early Golden Bantam (EGB) maize have shown that Erc1, a conserved effector with organ-specific virulence function in smut fungi, can promote intercellular expansion in the bundle sheath of plants (Ökmen et al., 2022). Another study found that an effector in maize Golden Bantam, Sts2 (Small tumor on seedlings 2), can act as a transcriptional activator and activate the expression of leaf developmental regulators to potentiate tumor formation (Zuo et al., 2023). These studies provide a molecular explanation for the successful colonization of *U. maydis* in maize, and our study sheds new light on this phenomenon from a chemical perspective. The increase in phytohormone contents in maize infected by *U. maydis* facilitated tumor expansion, and the high concentration of phenolic acid *p*HB was conducive to the germination of *U. maydis* spores. The two alkaloids MBOA and HMBOA were found to respond positively to *U. maydis* infection in this study but could not inhibit the infection of *U. maydis* in maize.

## 5 Conclusions

The impact of *U. maydis* infection on maize B73 is multifaceted. Although most of the specialized metabolites showing increased concentrations following *U. maydis* infection have certain defense capabilities, they did not achieve any defensive effects and were not correlated with the infecting *U. maydis*. The upregulation of various compounds in maize B73 following infection with *U. maydis* may be beneficial to the fungus. For example, the observed increase in phytohormones following infection with *U. maydis* may help the rapid growth of *U. maydis* infection sites into tumors in the short term. Furthermore, *p*HB, which is not specifically associated with *U. maydis* infection, was found to promote the growth of *U. maydis* spores at high concentrations. An increase in levels of *p*HB therefore promotes the colonization of maize B73 by *U. maydis*. In general, a series of “actions” of *U. maydis* after inoculating maize B73 indicate that fungal endophytes can promote plant growth and resist biological stress at the same time, which has been confirmed in recent years. Finally, the combined effect of these various influences reached the goal of successfully colonizing *U. maydis* on maize B73 plants. At this point, the adaptation mechanism of *U. maydis* to specialized metabolites in maize B73 has been more comprehensively explained, indicating that *U. maydis* can alter the balance in the growth-defense trade-off in maize B73.

## Data availability statement

The datasets presented in this study can be found in online repositories. The names of the repository/repositories and accession number(s) can be found in the article/**Supplementary Material**.

## Author contributions

XG: Data curation, Formal analysis, Investigation, Methodology, Software, Validation, Visualization, Writing – original draft. ZY: Data curation, Investigation, Writing – original draft. JZ: Data curation, Investigation, Methodology, Writing – original draft. JH: Conceptualization, Formal analysis, Funding acquisition, Methodology, Project administration, Resources, Visualization, Writing – review & editing. SL: Conceptualization, Funding acquisition, Methodology, Validation, Visualization, Writing – original draft, Writing – review & editing.
