# Supplementary material for: Adaptation of Ustilago maydis to phenolic and alkaloid responsive metabolites in maize B73
Source: Front Plant Sci. 2024 Jul 19;15:1369074. doi: 10.3389/fpls.2024.1369074 (PMC11294074; doi:10.3389/fpls.2024.1369074)
Supplement: Supplementary file 1 [file DataSheet_1.docx]

Supplementary Material

Adaptation of *Ustilago maydis* to phenolic and alkaloid responsive metabolites in maize B73

**Xuanyue Guo, Zhen Yang, Jinjin Zhang, Juan Hua*, Shihong Luo***

Engineering Research Center of Protection and Utilization of Plant Resources, College of Bioscience and Biotechnology, Shenyang Agricultural University, Shenyang, 110866, Liaoning Province, China

*** Correspondence:** Dr. Juan Hua huajuan@syau.edu.cn; Prof. Shihong Luo luoshihong@syau.edu.cn (http://orcid.org/0000-0003-3500-3466)

# Supplementary Note

## Correlation analyses between maize seedling growth indexes and the contents of phenolic acids or alkaloids in roots.

Correlation analyses were conducted between indicators representing the growth of maize B73 seedlings (length and weight) and alkaloid metabolites in the roots (Supplementary Figure S1A, B), and between growth indicators and the levels of phenolic acid metabolites (Supplementary Figure S1C, D).

No significant correlation was found in maize B73 seedling roots between alkaloids and growth indexes of maize B73 in either the inoculation group or the mechanical damage group (Figure S1A, B). Moreover, there was not association between MBOA, HMBOA, DMBOA, or NMC with the growth indices of maize B73 in either group. However, an association between DIBOA and length and weight of roots was found in the mechanical damage group, with the correlation coefficients being *r* = -0.88 (*p* < 0.001) and *r* = -0.74 (*p* < 0.001), and these were *r* = -0.07 and *r* = -0.35, respectively, in the inoculation group. BOA was significantly negatively correlated with growth indexes in the inoculation group but this association was weakened in the mechanical damage group.

In the roots of maize B73 seedlings, there was a negative correlation between phenolic compounds and the growth indexes of maize B73 in both the inoculation and mechanical damage groups (Supplementary Figure S1C, D). MeP, HBA, and VAE were negatively correlated with the length and weight of maize B73 in the inoculation group, and also negatively correlated with these indexes in the mechanical damage group. The correlation coefficient between *p*HB and weight in the inoculation group was *r* = -0.48 (*p* < 0.05), and *r* = -0.62 in the mechanical damage group (*p* < 0.01). MHPP was not associated with the growth indicators of maize B73 in either group.

# Supplementary Figures and Tables

## Supplementary Figures


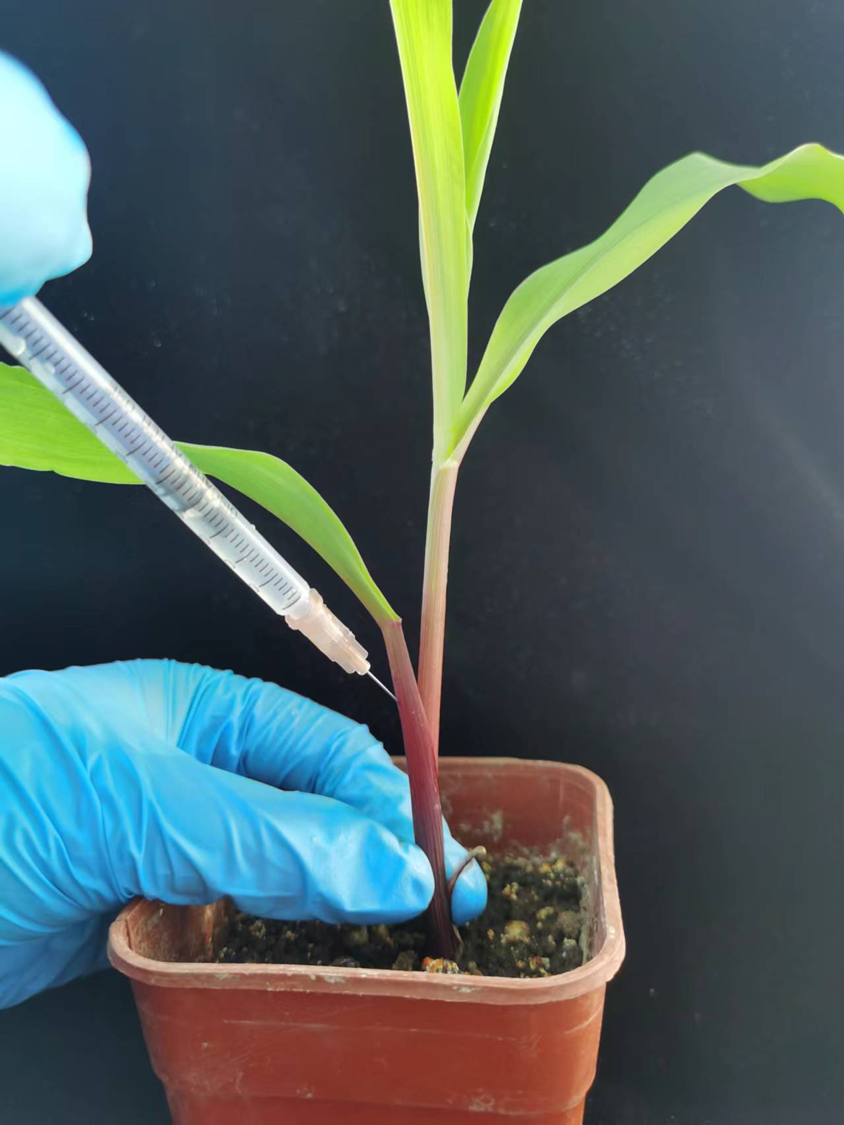


**Supplementary Figure 1.** Operation diagram of injecting spore suspension into maize B73.


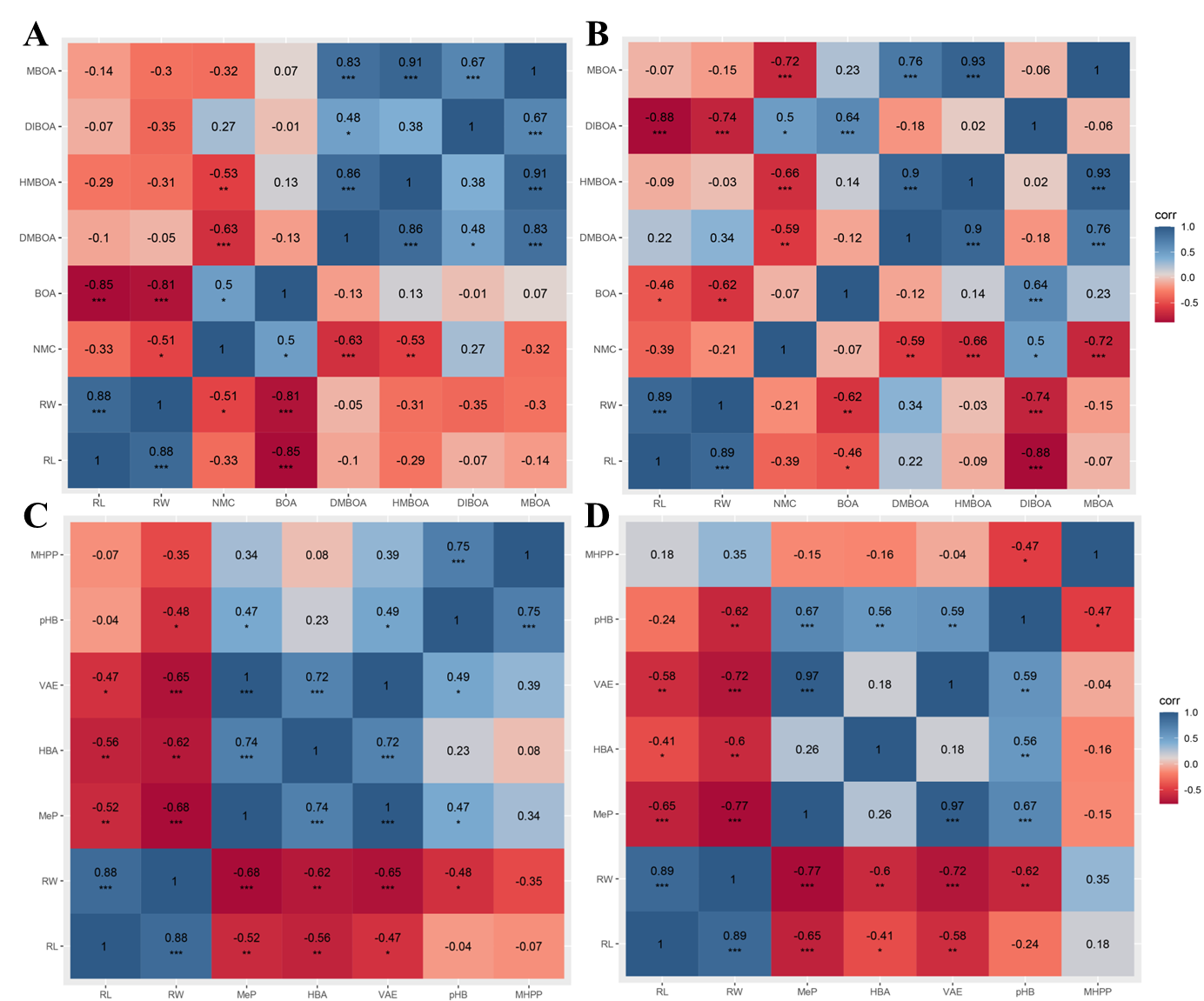
**Supplementary Figure 2.** Correlation analysis between maize root growth indexes and specialized metabolites. **(A)** Correlation analysis between length, weight, and alkaloids of the inoculation group. **(B)** Correlation analysis between length, weight, and defense alkaloids of the mechanical damage group. **(C)** Correlation analysis between length, weight, and phenolic acids of the inoculation group. **(D)** Correlation analysis between length, weight, and phenolic acids of the mechanical damage group. RL: Root Length; RW: Root Weight. The blue and red squares represent positive and negative correlations respectively. One, two, or three asterisks (*, **, or ***) indicate significant differences (at *p* < 0.05, *p* < 0.01, or *p* < 0.001, respectively) between the controls and the other treatments at the same time, as determined using Student *t*-tests.

## Supplementary Tables

**Supplementary Table S1.** Multi-reaction monitoring mode (MRM) conditions for compounds analyzed using UPLC-MS/MS.

| Specialized metabolites | Analyte | Mode | MRM transition |
| --- | --- | --- | --- |
| Phytohormones | IAA | ESI+ | 176.00>130.00 |
|  | 6BA | ESI+ | 225.95>148.25 |
|  | ABA | ESI- | 263.10>153.10 |
|  | SA | ESI- | 137.20>93.20 |
|  | GA_1_ | ESI- | 347.05>229.10 |
|  | GA_3_ | ESI- | 345.05>143.05 |
|  | GA_4_ | ESI- | 331.05>213.10 |
|  | GA_7_ | ESI- | 329.20>223.20 |
|  | GA_9_ | ESI- | 315.05>271.00 |
|  | GA_12_ | ESI- | 331.05>201.05 |
|  | GA_19_ | ESI- | 361.05>133.00 |
|  | GA_24_ | ESI- | 345.05>213.15 |
|  | GA_53_ | ESI- | 347.10>189.10 |
|  | IPA | ESI+ | 189.85>130.10 |
|  | *t*Z | ESI+ | 220.10>136.05 |
| Phenolic acids | HBA | ESI- | 121.20>92.05 |
|  | MeP | ESI- | 151.15>92.05 |
|  | VAE | ESI- | 151.25>91.90 |
|  | *p*HB | ESI- | 136.90>92.95 |
|  | MHPP | ESI- | 178.85>117.15 |
|  | Syringic acid | ESI- | 196.95>182.00 |
|  | Ferulic acid | ESI- | 192.95>134.15 |
|  | *p*-Coumaric acid | ESI- | 162.85>119.20 |
|  | Caffeic acid | ESI- | 179.05>134.95 |
|  | Benzoic acid | ESI- | 121.20>62.10 |
|  | 4-Hydroxybenzoic acid | ESI- | 137.00>93.15 |
| Alkaloids | MBOA | ESI- | 164.20>121.10 |
|  | HMBOA | ESI- | 194.00>179.00 |
|  | DMBOA | ESI+ | 196.10>181.30 |
|  | NMC | ESI- | 181.15>112.90 |
|  | DIBOA | ESI- | 163.85>108.00 |
|  | BOA | ESI- | 133.90>42.00 |

**Supplementary** **Table S2.** Elution procedure for the analytes.

| **Specialized metabolites** | **Time (min)** | **B (acetonitrile) phase concentration (%)** |
| --- | --- | --- |
| Phytohormones and  Phenolic acids | 0 | 5 |
|  | 12 | 95 |
|  | 14 | 95 |
|  | 15 | 5 |
|  | 18 | 5 |
| Alkaloids | 0 | 5 |
|  | 12 | 95 |
|  | 13 | 95 |
|  | 14 | 5 |
|  | 17 | 5 |

**Supplementary Table S3.** Calibration equations for the analytes.

| Specialized metabolites | Analyte | Calibration equations | R^2^ |
| --- | --- | --- | --- |
| Phytohormones | IAA | Y = (2.03×10^-7^)X-0.0450 | 0.9993 |
|  | 6BA | Y = (2.06×10^-5)^X-0.0172 | 0.9985 |
|  | ABA | Y = (8.52×10^-6^)X+0.0083 | 0.9987 |
|  | SA | Y = (5.77×10^-8^)X-0.0038 | 0.9900 |
|  | GA_1_ | Y=(1.99×10^-5)^X-0.0102 | 0.9935 |
|  | GA_3_ | Y = (3.25×10^-6^)X-0.0030 | 0.9994 |
|  | GA_4_ | Y = (4.99×10^-6^)X-0.0077 | 0.9986 |
|  | GA_7_ | Y=(2.35×10^-5^)X-0.0839 | 0.9933 |
|  | GA_9_ | Y=(9.54×10^-7^)X-0.0088 | 0.9929 |
|  | GA_12_ | Y=(2.12×10^-5^)X-0.0023 | 0.9933 |
|  | GA_19_ | Y = (2.13×10^-5^)X+0.0034 | 0.9972 |
|  | GA_24_ | Y=(2.92×10^-5^)X-0.0011 | 0.9952 |
|  | GA_53_ | Y = (6.12×10^-6^)X+0.0002 | 0.9950 |
|  | IPA | Y = (1.31×10^-7^)X+0.0257 | 0.9984 |
|  | *t*Z | Y = (1.17×10^-7^)X-0.0291 | 0.9984 |
| Phenolic acids | HBA | Y = (1.29×10^-7^)X-0.0190 | 0.9989 |
|  | MeP | Y=(8.15×10^-5^)X+0.0066 | 0.9987 |
|  | VAE | Y=(8.11×10^-5^)X-0.0083 | 0.9968 |
|  | *p*HB | Y=(1.96×10^-6^)X-0.0200 | 0.9986 |
|  | MHPP | Y=(2.7010^-6^)X-0.00003 | 0.9929 |
|  | Syringic acid | Y = (2.82×10^-3^)X-2.36153 | 0.9987 |
|  | Ferulic acid | Y = (3.61×10^-5^)X+0.0078 | 0.9988 |
|  | *p*-Coumaric acid | Y = (1.52×10^-7^)X-0.0495 | 0.9982 |
|  | Caffeic acid | Y = (1.41×10^-5^)X-2.11041 | 0.9993 |
|  | Benzoic acid | Y = (9.86×10^-4^)X+0.0959 | 0.9982 |
|  | 4-Hydroxybenzoic acid | Y = (7.22×10^-6^)X-0.0357 | 0.9989 |
| Alkaloids | MBOA | Y = (3.12×10^-4^)X-0.1208 | 0.9984 |
|  | HMBOA | Y = (8.98×10^-5^)X-0.0066 | 0.9935 |
|  | DMBOA | Y = (7.81×10^-3^)X-0.0390 | 0.9985 |
|  | NMC | Y = (1.07×10^-6^)X-0.7849 | 0.9933 |
|  | DIBOA | Y = (5.06×10^-5^)X-0.0141 | 0.9972 |
|  | BOA | Y = (3.62×10^-4^)X-0.0167 | 0.9986 |
